# BLOOD BIOMARKERS OF NEURODEGENERATION ARE ASSOCIATED WITH SMARTPHONE-ASSESSED SUBJECTIVE COGNITIVE CONCERNS

**DOI:** 10.1093/geroni/igae098.0732

**Published:** 2024-12-31

**Authors:** Ángel García De La Garza, Cuiling Wang, Jacqueline Mogle, Carol Derby, Richard Lipton, Laura Rabin, Mindy Katz

**Affiliations:** Albert Einstein College of Medicine, Bronx, New York, United States; Albert Einstein College of Medicine, Bronx, New York, United States; Clemson University, Clemson, South Carolina, United States; Albert Einstein College of Medicine, Bronx, New York, United States; Albert Einstein College of Medicine, Bronx, New York, United States; Brooklyn College and The Graduate Center of CUNY, Brooklyn, New York, United States; Albert Einstein College of Medicine, Bronx, New York, United States

## Abstract

The association between blood-based biomarkers of Alzheimer’s Disease (BBBs) and daily diary smartphone assessment of subjective cognitive concerns (SCC) is unexplored. In the Einstein Aging Study, we collected digital diary information on 18 types of cognitive lapses per day over a 14-day period on 271 participants (mean age 77.50, SD 4.90, 66.8% Female, 47.6% Non-Hispanic-White, 40.2% Non-Hispanic-Black, 12.1% Hispanic). BBBs investigated included β-amyloid (Aβ40, Aβ42), the Aβ42:Aβ40 ratio, pTau181, neurofilament light (NfL), and glial fibrillary acidic protein (GFAP). Daily diary SCC was analyzed by calculating the average daily number of lapses related to memory (12 items, mean = 0.99, SD = 0.97), the average daily number of lapses related to executive functioning (6 items, mean = 0.53, SD = 0.74), and the daily average number of all cognitive lapses (18 items, mean = 1.53, SD = 1.59), where higher scores indicated greater self-reported cognitive difficulties. Utilizing separate linear regression, we assessed the association between BBB levels and cognitive lapses, adjusting for age, sex, ethnicity, depression, and years of education. Findings indicate that, on average, for every 1 standard deviation increase in blood levels of pTau181, participants reported an additional 0.21 additional cognitive lapses in the memory domain (p< 0.001) and 0.11 in the executive functioning domain (p=0.012) for a total of 0.32 daily cognitive lapses (p< 0.001). No significant associations were identified between Aβ40, Aβ42, Aβ42:Aβ40 ratio, NfL, GFAP, and daily diary SCC. Captured in this manner, SCC may serve as a scalable indicator of AD pathology, progression, and monitoring the disease.

